# Methotrexate in relatively contraindicated ectopic pregnancies: who benefits from treatment?

**DOI:** 10.1007/s00404-026-08501-4

**Published:** 2026-07-11

**Authors:** Gülhan Özüm, Hakan Güraslan, Nurşen Kurtoğlu, Levent Deniz

**Affiliations:** 1 Clinic of Gynecology, University of Health Sciences, Istanbul Bağcılar Training and Research Hospital, Istanbul, Turkey; 2 Department of Medical Biochemistry, University of Health Sciences, Istanbul Training and Research Hospital, Istanbul, Turkey

**Keywords:** Ectopic pregnancy, Methotrexate, Relative contraindication, High β-hCG level, Free pelvic fluid, Treatment outcome

## Abstract

**Objective:**

This study aimed to evaluate the efficacy of methotrexate (MTX) therapy and identify clinical and laboratory predictors of treatment failure, particularly in relatively contraindicated cases.

**Materials and methods:**

This retrospective cohort study included 225 hemodynamically stable patients with ectopic pregnancy treated with MTX between January 2019 and December 2024 at a tertiary university hospital. Relatively contraindicated cases were defined as having one or more of the following: β-hCG ≥ 5000 IU/L, ectopic mass ≥ 4 cm, fetal cardiac activity, or pelvic free fluid ≥ 5 cm. 58 patients met these criteria, while 167 were MTX-indicated. All patients received a single-dose intramuscular MTX protocol (50 mg/m^2^ on day 0), and serum β-hCG levels were measured on days 0, 4, and 7. Treatment success was defined as a decline of β-hCG to < 5 mIU/mL without surgical intervention. Predictive factors for treatment failure were analyzed using logistic regression. Based on the identified independent predictors, a preliminary scoring model was constructed to explore risk stratification.

**Results:**

The overall MTX success rate was 85.8%. Success was significantly higher in the indicated group (92.8%) than in the relatively contraindicated group (65.5%). In the latter, treatment failure was significantly associated with higher baseline β-hCG levels and the pelvic free fluid ≥ 5 cm in the pouch of douglas. Multivariate analysis identified a significant association between these factors and MTX failure (β-hCG: OR 7.44, p < 0.001; free fluid: OR 4.10, p = 0.022). Other parameters, including fetal cardiac activity, yolk sac presence, adnexal mass size, and patient age, did not show a significant relationship in this model. The median time to surgical intervention in failed cases was 7 days.

**Conclusion:**

Single-dose MTX remains a safe and effective option for carefully selected patients with relatively contraindicated ectopic pregnancies, achieving a 65.5% success rate. High baseline β-hCG and pelvic free fluid may serve as predictors of treatment failure, but do not preclude medical management when close monitoring is ensured. The proposed scoring system should be considered exploratory and requires external validation before clinical implementation. Further prospective studies are needed to confirm these findings and better define candidates for MTX therapy in high-risk cases.

**Supplementary Information:**

The online version contains supplementary material available at 10.1007/s00404-026-08501-4.

## What does this study add to the clinical work?

**Table:** 

In carefully selected hemodynamically stable patients with ectopic pregnancy and relative contraindications, single-dose methotrexate achieved a clinically meaningful success rate. Baseline β-hCG levels and pelvic free fluid ≥ 5 cm may help stratify the risk of treatment failure and support individualized management decisions.	

## Introduction

Ectopic pregnancy is characterized by the implantation of a fertilized ovum outside the uterine cavity and represents a significant complication during the first trimester of pregnancy [1–3]. Approximately 98% of cases are located in the fallopian tubes, while rarer sites include the ovary, uterine cornu, interstitial, intra-abdominal, or cervical regions [4, 5]. The incidence is approximately 2%; however, it can rise up to 20% among women with a previous history of ectopic pregnancy or tubal surgery [3, 6, 7]. It remains one of the leading causes of maternal morbidity and mortality [8–10].

Early diagnosis and treatment are crucial for preserving fertility and reducing the risk of morbidity [6, 11]. However, the clinical manifestations are often nonspecific and may range from asymptomatic cases to life-threatening conditions [12]. Transvaginal ultrasonography (TVS) and serum beta-human chorionic gonadotropin (β-hCG) measurements are the main diagnostic tools [3, 13]. The finding of an empty uterine cavity with a β-hCG level ≥ 1500 IU/L increases the diagnostic accuracy [2]. According to current guidelines, surgical management is preferred for hemodynamically unstable patients; however, there is no consensus regarding medical treatment approaches [5, 14]. Nevertheless, medical therapy is prioritized for hemodynamically stable patients. Methotrexate (MTX) remains the cornerstone agent in the medical management of ectopic pregnancy and was first introduced for this purpose in the early 1980 s [4, 8]. As a folate antagonist, it inhibits DNA synthesis and is particularly effective in rapidly dividing cells [15–17]. MTX treatment protocols include single-, two-, and multiple-dose regimens [2, 8, 14].

A subset of patients remains without a clear consensus regarding treatment despite hemodynamic stability and is considered relatively contraindicated for medical therapy. This group included cases with at least one of the following: β-hCG ≥ 5000 mIU/mL, ectopic mass ≥ 4 cm, fetal cardiac activity (FCA), or abdominal free fluid ≥ 5 cm [18]. While some studies advocate surgery as first-line management, our study aimed to evaluate a noninvasive approach because of limited literature. We retrospectively assessed MTX efficacy in ectopic pregnancies and identified clinical and laboratory factors associated with treatment failure, focusing on relatively contraindicated cases.

## Materials and methods

### Study design and participants

This retrospective cohort study was conducted at a tertiary hospital (January 2019–December 2024) and included 225 patients with ectopic pregnancy confirmed by serum β-hCG and TVS. Of these, 58 were classified as MTX relatively contraindicated and 167 as MTX indicated. The relatively contraindicated group included patients who declined surgery for fertility reasons and opted for MTX therapy. Ethical approval was obtained (No: 2025/05/01/044), and the study adhered to the principles of the Declaration of Helsinki. Informed consent was waived due to the retrospective design of the study by the Institutional Ethics Committee. The inclusion criteria were hemodynamically stable patients with ectopic pregnancy confirmed by TVS and/or serial β-hCG who received at least one dose of MTX. The exclusion criteria were as follows: expectant or primary surgical management, absolute MTX contraindications (immunodeficiency, hemodynamic instability, hypersensitivity, breastfeeding, renal/hepatic failure, peptic ulcer, or pulmonary disease), and interstitial or scar ectopic pregnancy. Diagnosis was based on β-hCG ≥ 1500 mIU/mL without an intrauterine gestational sac, with TVS evidence of an ectopic focus (gestational sac, yolk sac, FCA) or inadequate serial β-hCG rise (< 66% increase or plateau in 48 h).

### Treatment protocol

All patients received a single-dose MTX protocol (50 mg/m2, intramuscularly (IM) on day 0), and serum β-hCG levels were measured on days 0, 4, and 7. Treatment success was defined as β-hCG < 5 mIU/mL without surgery; cases requiring surgery were considered failures. Patients who received a second dose of MTX were included in the outcome analyses according to their final treatment response (successful vs. failed), regardless of the number of doses administered. Treatment response was evaluated according to the standard single-dose protocol using serial β-hCG measurements on days 4 and 7. An adequate response was defined as a ≥ 15% decrease in β-hCG levels between days 4 and 7. An inadequate response was defined as a < 15% decrease in β-hCG levels and/or a plateau or increase in β-hCG levels [19]. A second dose of MTX was not routinely administered and was applied selectively according to predefined response criteria. All patients were hospitalized and monitored clinically and biochemically, as well as via TVS. Stable patients in the indicated group were discharged, whereas those in the relatively contraindicated group remained hospitalized for further observation. No patient discontinued treatment owing to adverse effects, and no serious complications occurred. Responders were followed up weekly until β-hCG levels were < 5 mIU/mL. Surgery was performed in cases of MTX treatment failure, clinical instability, or suspected tubal rupture. In this cohort, all patients classified as treatment failure underwent surgical intervention, and tubal rupture was confirmed intraoperatively in every case. No medically managed treatment failures were observed, and salpingectomy was performed in all surgically treated patients. While tubal rupture was identified in all treatment failure cases, treatment failure remained the predefined outcome for all statistical analyses.

### Data collection and measurements

**Fig. 1:**
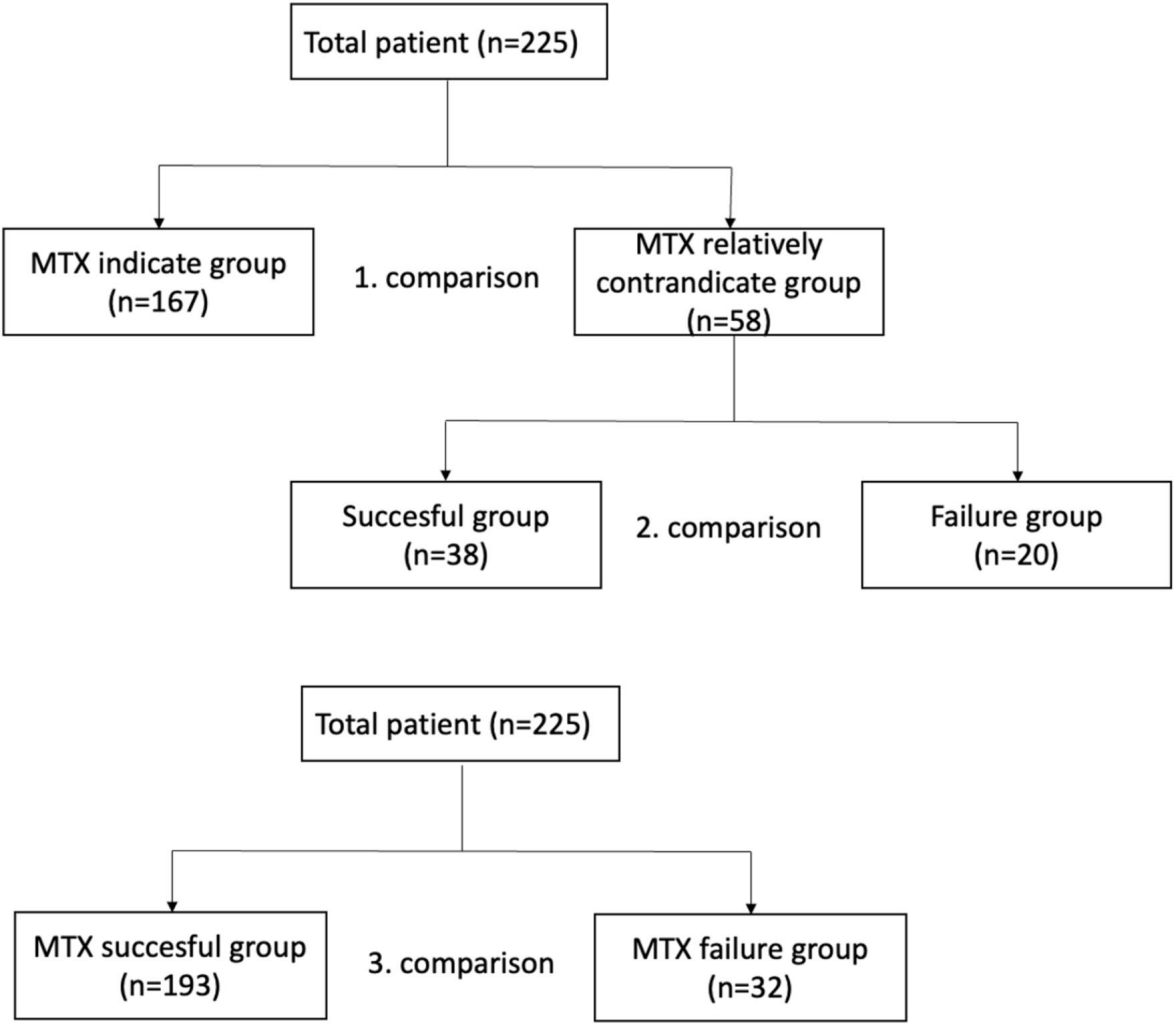
Schematic representation of the analysis flow

Demographic and clinical data (age, gravidity, gestational age, prior ectopic pregnancy, and presenting symptoms), ultrasonographic findings (endometrial thickness, ectopic mass size, pelvic free fluid, yolk sac, and FCA), and laboratory results were documented. The 48 h pre-treatment β-hCG increase, β-hCG levels on days 0, 4, and 7, number of MTX doses, treatment response, need for surgery, pathological findings, interval between MTX and tubal rupture, and hospital stay were evaluated. Serum β-hCG was measured by electrochemiluminescence immunoassay. The ectopic mass size was recorded as the maximum diameter on TVS; when indistinguishable from a hematoma, the total complex mass was measured. The patients were analyzed at three levels (Fig. 1):

- 1. Comparison between the MTX-indicated and relatively contraindicated groups.
- 2. Comparison between the relatively contraindicated successful and relatively contraindicated failure groups.
- 3. Comparison between successful and failure cases in the entire cohort (n = 225);

### Statistical analysis

Data distribution was assessed using the Shapiro–Wilk test. Normally distributed variables are presented as mean ± standard deviation, and non-normally distributed variables as median (25th–75th percentile). Categorical variables were analyzed with the Chi-square or Fisher’s exact test when appropriate; Fisher’s test was applied if > 20% of cells had expected counts < 5. For two-group comparisons, the Student’s t-test was used for parametric variables and the Mann–Whitney U test for nonparametric variables. The optimal baseline β-hCG cutoff for predicting MTX treatment failure was determined from ROC curve analysis using the Youden index (sensitivity + specificity − 1). Independent risk factors for treatment failure were evaluated using binary logistic regression. Analyses were performed in SPSS version 26.0 (IBM Corp., Armonk, NY, USA) and MedCalc Statistical Software version 23.4.5 (MedCalc Software Ltd, Ostend, Belgium), with p < 0.05 considered significant. Calibration was examined by comparing observed and predicted treatment failure probabilities across score categories and by calculating the calibration intercept, calibration slope, Brier score, and Hosmer–Lemeshow goodness-of-fit statistic. Predicted probabilities were derived from a logistic regression model based on the proposed score. Calibration plots were generated to visually assess agreement between predicted and observed event rates.

## Results

A total of 225 patients with ectopic pregnancy who received MTX treatment were included in the study. The overall treatment success rate was 85.8%, with rates of 92.8% in the indication group and 65.5% in the relative contraindication group (Table 1). Among all patients, 31 (13.8%) received two consecutive single-dose methotrexate treatments, of whom 13 (41.9%) belonged to the relative contraindication group. In this group, the mass size, volume of fluid in the douglas pouch, endometrial thickness, rates of yolk sac, FCA, vaginal spotting, pelvic pain, length of hospital stay, and β-hCG levels on days 0, 4, and 7 were significantly higher than those in the indication group. However, no significant differences were observed between the groups in terms of the percentage changes in β-hCG levels (pre-treatment increase, day 4 vs. day 0, day 7 vs. day 4, and day 7 vs. day 0).

**Table 1:** Comparison of demographic, clinical and laboratory characteristics between the groups

Variable	Total (n = 225)	Indication (n = 167)	Relative contraindication (n = 58)	P-value
Age (years)	32.0 (28.0–37.0)	32.0 (28.0–37.0)	32.0 (25.0–35.0)	0.327
Gravidity (n)	3.00 (2.00–4.00)	3.00 (2.00–4.00)	3.00 (2.00–4.00)	0.630
Gestational age (weeks)	6.14 (5.00–7.07)	6.07 (4.57–7.29)	6.29 (5.71–6.86)	0.765
CS history, n (%)	89 (39.6)	64 (38.3)	25 (43.1)	0.627
AB history, n (%)	78 (34.7)	58 (34.7)	20 (34.5)	0.973
EP history, n (%)	31 (13.8)	21 (12.6)	10 (17.2)	0.505
Mass size (mm)	13.0 (0.00–20.0)	11.0 (0.00–18.0)	16.0 (10.0–24.0)	< 0.001
Fluid in Douglas (cm)	0.00 (0.00–1.00)	0.00 (0.00–1.00)	0.50 (0.00–5.00)	< 0.001
Endometrial thickness (mm)	6.00 (6.00–10.3)	6.00 (6.00–9.00)	9.00 (6.00–13.0)	< 0.001
Yolk sac, n (%)	12 (5.33)	4 (2.40)	8 (13.8)	0.003
Revision curettage, n (%)	98 (43.6)	72 (43.1)	26 (44.8)	0.821
Symptoms, n (%)				
Vaginal bleeding	92 (40.9)	73 (43.7)	19 (32.8)	0.191
Pelvic pain	49 (21.8)	35 (21.0)	14 (24.1)	0.748
Vaginal bleeding and pelvic pain	46 (20.4)	27 (16.2)	19 (32.8)	0.012
Asymptomatic	38 (16.9)	32 (19.2)	6 (10.3)	0.180
Positive fetal heartbeat, n (%)	8 (3.56)	0 (0.00)	8 (13.8)	< 0.001
Number of MTX treatments	1.00 (1.00–1.00)	1.00 (1.00–1.00)	1.00 (1.00–1.00)	0.077
LOS (days)	8.00 (5.00–11.3)	7.00 (4.00–10.0)	9.50 (7.00–15.0)	< 0.001
Day-0 β-hCG (IU/L)	2044 (602.5–4105)	1478 (500.0–2562)	6036 (3417–9254)	< 0.001
Day-4 β-hCG (IU/L)	1442 (432.5–3290)	955.0 (331.5–2133)	3818 (1993–6935)	< 0.001
Day-7 β-hCG (IU/L)	969.0 (218.8–2494)	676.0 (192.0–1671)	2516 (933.8–5497)	< 0.001
β-hCG change%: day-4 vs. day-0	−9.94 (−40.2 to 14.3)	−6.83 (−37.2 to 14.6)	15.4 (−43.3 to 10.4)	0.440
β-hCG change%: day-7 vs. day-4	−28.9 (−50.1 to −13.5)	−26.3 (−46.2 to −12.5)	−35.7 (−55.4 to −19.9)	0.220
β-hCG change%: day-7 vs. day-0	−38.8 (−67.7 to −8.11)	−31.9 (−66.9 to −7.09)	−42.8 (−74.9 to −15.6)	0.184
Rate of β-hCG rise in 48 h (pre-treatment)	4.52 (−7.43 to 18.6)	4.68 (−4.88 to 18.6)	3.77 (−13.4 to 19.3)	0.548
Success of Treatment, n (%)	193 (85.8)	155 (92.8)	38 (65.5)	<0.001

P < 0.05: Statistically significant. *CS *cesarean section, *AB* abortion, *EP* ectopic pregnancy, *MTX* methotrexate, *LOS* length of stay in hospital, *β-hCG* beta-human chorionic gonadotropin

When the relative contraindication group was stratified into successful and failed subgroups according to treatment response, patients in the successful subgroup were older and had lower β-hCG levels on day 4. No significant differences were observed in the other β-hCG measurements or their percentage change (Table 2). The median interval between MTX administration and subsequent surgical intervention was 7 days (range, 1–30 days).

**Table 2:** Comparison of demographic, clinical and laboratory characteristics between the groups

Variable	Relative contraindication (n = 58)		P-value
	Failure (n = 20)	Success (n = 38)	
Age (years)	28.5 (24.0–33.0)	32.5 (29.0–37.0)	0.037
Gravidity (n)	2.50 (1.00–3.00)	3.00 (2.00–4.00)	0.124
Gestational age (weeks)	6.12 ± 1.31	6.15 ± 1.44	0.949
CS history, n (%)	8 (40.0)	17 (44.7)	0.946
AB history, n (%)	6 (30.0)	14 (36.8)	0.818
EP history, n (%)	5 (25.0)	5 (13.2)	0.290
Mass size (mm)	15.0 (12.5–23.5)	17.0 (9.00–24.0)	0.889
Mass size ≥ 40 mm, n (%)	2 (10.0)	5 (13.2)	0.726
Fluid in Douglas (cm)	2.50 (0.00–5.00)	0.00 (0.00–5.00)	0.840
Fluid in Douglas ≥ 5 cm, n (%)	6 (30.0)	10 (26.3)	0.765
Endometrial thickness (mm)	8.50 (6.00–12.5)	9.50 (6.00–13.0)	0.460
Visible yolk sac, n (%)	4 (20.0)	4 (10.5)	0.428
Revision curettage, n (%)	10 (50.0)	16 (42.1)	0.767
Symptoms, n (%)			
Vaginal bleeding	4 (20.0)	15 (39.5)	0.227
Pelvic pain	6 (30.0)	8 (21.1)	0.525
Vaginal bleeding and pelvic pain	8 (40.0)	11 (28.9)	0.577
Asymptomatic	2 (10.0)	4 (10.5)	0.950
Positive fetal heartbeat, n (%)	4 (20.0)	4 (10.5)	0.428
Number of MTX treatments	1.00 (1.00–1.00)	1.00 (1.00–1.00)	0.926
LOS (days)	9.00 (7.50–18.0)	10.5 (7.00–14.0)	0.550
Day-0 β-hCG (IU/L)	6228 (4592–11,086)	5700 (2105–7897)	0.169
Day-4 β-hCG (IU/L)	5817 (2891–9684)	3408 (1179–6037)	0.046
Day-7 β-hCG (IU/L)	3300 (2090–7032)	1999 (616.0–5456)	0.131
β-hCG change%: day-4 vs. day-0	−8.75 (−26.9 to 24.9)	−16.5 (−44.3 to 4.98)	0.177
β-hCG change%: day-7 vs. day-4	−32.7 (−52.4 to −2.02)	−37.4 (−58.4 to −20.3)	0.610
β-hCG change%: day-7 vs. day-0	−40.2 (−62.2 to −11.8)	−48.0 (−76.6 to −23.2)	0.392
Rate of β-hCG rise in 48 h (pre-treatment)	6.24 (−2.10 to 25.3)	2.78 (−16.6 to 9.35)	0.185

P < 0.05: Statistically significant. *CS* cesarean section, *AB* abortion, *EP* ectopic pregnancy, *MTX* methotrexate, *LOS* length of stay in hospital, *β-hCG* beta-human chorionic gonadotropin

All 32 patients classified as treatment failure underwent surgical intervention according to the predefined study outcome definition, and no patients were classified as treatment failure without subsequent surgical management. Intraoperatively, tubal rupture was confirmed in all cases, and salpingectomy was performed. Histopathological examination revealed findings consistent with tubal ectopic pregnancy. In this group, the presence and amount of fluid in the douglas pouch, rate of FCA, length of hospital stay, and β-hCG levels on days 0, 4, and 7 were significantly higher than those in the successful treatment group. However, no significant differences were observed in the percentage changes of β-hCG (Table 3).

**Table 3:** Comparison of demographic, clinical and laboratory characteristics between the groups

Variable	Failure (n = 32)	Success (n = 193)	P-value
Age (years)	30.5 (24.5–33.5)	32.0 (28.0–37.0)	0.113
Gravidity (n)	3.00 (2.00–3.00)	3.00 (2.00–4.00)	0.345
Gestational age (weeks)	6.14 (5.57–7.15)	6.14 (4.93–7.07)	0.701
CS history, n (%)	13 (40.6)	76 (39.4)	0.894
AB history, n (%)	9 (28.1)	69 (35.8)	0.523
EP history, n (%)	6 (18.8)	25 (13.0)	0.406
Mass size (mm)	14.0 (10.0–20.0)	12.0 (0.00–20.0)	0.125
Mass size ≥ 40 mm, n (%)	2 (6.30)	5 (2.60)	0.261
Fluid in Douglas (cm)	1.50 (0.00–3.00)	0.00 (0.00–1.00)	0.006
Fluid in Douglas ≥ 5 cm, n (%)	6 (18.8)	10 (5.20)	0.015
Endometrial thickness (mm)	8.00 (6.00–11.5)	6.00 (6.00–10.0)	0.303
Visible yolk sac, n (%)	4 (12.5)	8 (4.10)	0.073
Revision curettage, n (%)	15 (46.9)	83 (43.0)	0.829
Symptoms, n (%)			
Vaginal bleeding	9 (28.1)	83 (43.0)	0.164
Pelvic pain	11 (34.4)	38 (19.7)	0.102
Vaginal bleeding and pelvic pain	9 (28.1)	37 (19.2)	0.354
Asymptomatic	3 (9.40)	35 (18.1)	0.332
Positive fetal heartbeat, n (%)	4 (12.5)	4 (2.10)	0.016
Number of MTX treatments	1.00 (1.00–1.00)	1.00 (1.00–1.00)	0.379
LOS (days)	9.00 (7.00–17.5)	8.00 (4.00–11.0)	0.006
Day-0 β-hCG (IU/L)	4238 (2397–8353)	1649 (530.0–3302)	< 0.001
Day-4 β-hCG (IU/L)	4647 (2246–6564)	1039 (361.0–2849)	< 0.001
Day-7 β-hCG (IU/L)	3194 (1199–5541)	800.0 (199.5–2014)	< 0.001
β-hCG change%: day-4 vs. day-0	− 1.87 (− 26.8 to 29.3)	− 10.8 (− 42.6 to 11.8)	0.088
β-hCG change%: day-7 vs. day-4	− 31.7 (− 52.1 to − 2.16)	− 27.3 (− 49.4 to − 14.1)	0.935
β-hCG change%: day-7 vs. day-0	− 28.2 (− 56.5 to 6.51)	− 39.5 (− 68.7 to –8.60)	0.258
Rate of β-hCG rise in 48 h (pre-treatment)	7.03 (− 2.68 to 25.9)	3.20 (− 8.39 to 18.1)	0.147

P < 0.05: Statistically significant. *CS* cesarean section, *AB* abortion, *EP* ectopic pregnancy, *MTX* methotrexate, *LOS* length of stay in hospital, *β-hCG* beta-human chorionic gonadotropin

**Fig. 2:**
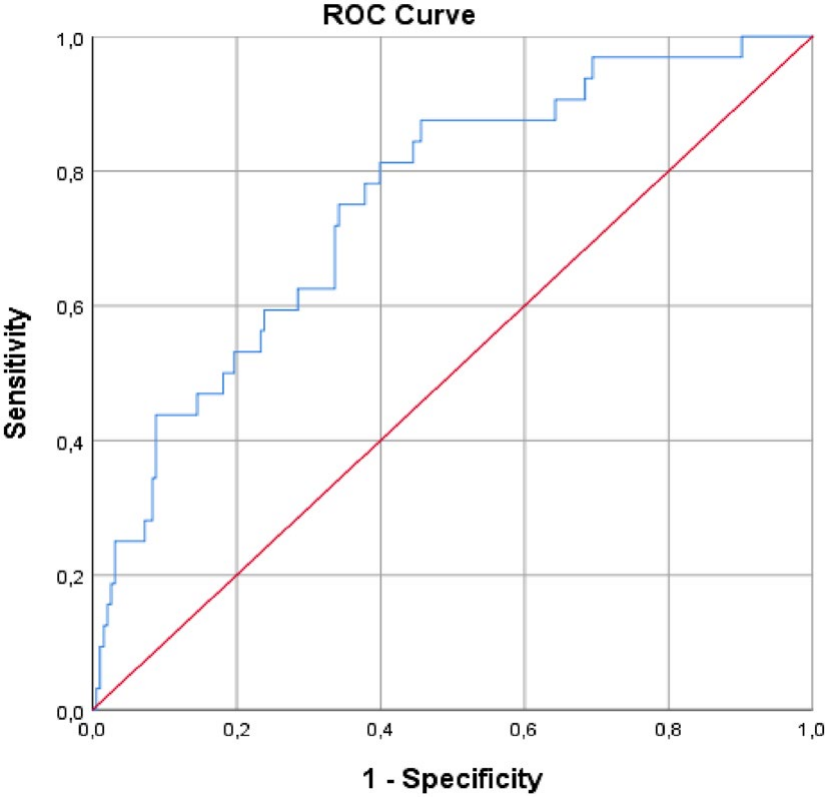
Receiver operating curve analysis of the relationship between initial β-hCG level and MTX treatment failure

In the ROC analysis performed to predict treatment failure, the area under the curve (AUC) for baseline (day 0) β-hCG was 0.754 (95% CI 0.692–0.808). The optimal cutoff value for baseline β-hCG was 1968 IU/L. At this threshold, the treatment success rate was 96.3%, with a sensitivity of 87.5% and a specificity of 54.4% (p < 0.001; Fig. 2). In addition, treatment success was further evaluated across stratified categories of baseline β-hCG levels for descriptive purposes. Accordingly, the treatment success rate was 95.5% in patients with baseline β-hCG levels < 1999 IU/L, while it was 50.0% in those with levels ≥ 10,000 IU/L (Table 4).

**Table 4:** MTX treatment success by stratified baseline β-hCG levels in the total cohort

Day-0 β-hCG (IU/L)	Failure (n)	Success (n)	Success Rate (%)
< 1999	5	105	95.5
2000–4999	13	62	82.7
5000–9999	8	20	71.4
≥ 10,000	6	6	50.0

*β-hCG* beta-human chorionic gonadotropin

In the univariate analysis, high baseline β-hCG levels (OR = 8.35; 95% CI 2.82–24.73; p < 0.001), presence of FCA (OR = 6.75; 95% CI 1.60–28.54; p = 0.009), and pelvic free fluid ≥ 5 cm in the pouch of douglas (OR = 4.22; 95% CI 1.42–12.59; p = 0.010) were found to be associated with treatment failure. In the multivariate analysis, conducted in the entire study cohort (n = 225), high β-hCG levels (OR = 7.44; 95% CI 2.46–22.50; p < 0.001) and pelvic free fluid ≥ 5 cm in the pouch of douglas (OR = 4.10; 95% CI 1.23–13.65; p = 0.022) were independently associated with treatment failure (Table 5). The events-per-variable (EPV) for the final multivariable model was approximately 10.7.

**Table 5:** Binary logistic regression analysis for predictors of MTX treatment failure in the entire study cohort

Variables	Univariate (Unadjusted)	Multivariate (Adjusted)	Variables	Univariate (Unadjusted)
	OR (95%CI)	P-value		OR (95%CI)
Initial β-hCG > 1968 IU/L	8.35 (2.82–24.73)	< 0.001	Initial β-hCG > 1968 IU/L	8.35 (2.82–24.73)
Positive fetal heartbeat	6.75 (1.60–28.54)	0.009	Positive fetal heartbeat	6.75 (1.60–28.54)
Fluid in Douglas ≥ 5 cm	4.22 (1.42–12.59)	0.010	Fluid in Douglas ≥ 5 cm	4.22 (1.42–12.59)

*OR* odds ratio, *β-hCG* beta-human chorionic gonadotropin

In our study, to assess the potential influence of second-dose MTX administration, a sensitivity analysis restricted to patients receiving a single-dose MTX regimen was performed after excluding those who received a second dose. The findings were generally similar to those of the primary analysis, with baseline β-hCG levels and pelvic free fluid ≥ 5 cm remaining statistically significant independent predictors of treatment failure. No additional statistically significant predictors were identified (Suppl Table 1–2).

**Fig. 3:**
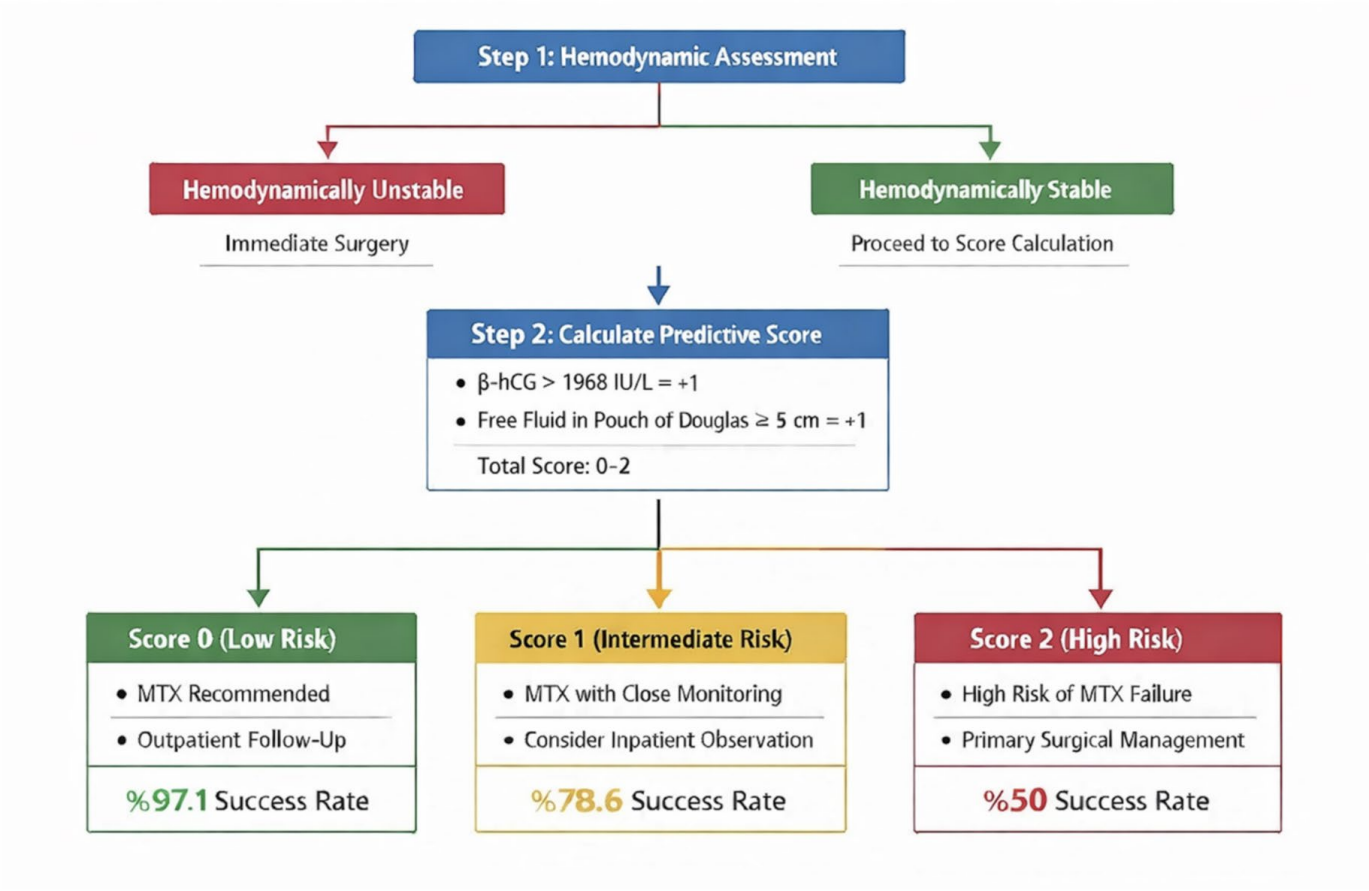
Exploratory risk stratification framework based on the proposed scoring model

Based on the multivariate analysis, two independent predictors of MTX treatment failure were identified: baseline β-hCG > 1968 IU/L and pelvic free fluid ≥ 5 cm. A simple predictive scoring system was developed by assigning 1 point for each predictor, generating a 0–2 score (Fig. 3). As shown in Table 6, treatment success decreased with increasing score: patients with a score of 0 had the highest success rate (100/103, 97.1%), those with a score of 1 had a success rate of 88/112 (78.6%), and patients with a score of 2 had the lowest success rate at 5/10 (50.0%). Corresponding treatment failure rates were 2.9%, 21.4%, and 50.0%, respectively (P < 0.001). ROC analysis demonstrated a moderate discriminative performance of the score for predicting treatment failure, with an AUC of 0.738 (95% CI 0.676–0.794, p < 0.001). Using a cutoff value of ≥ 1, the score achieved a sensitivity of 90.6% and a specificity of 51.8% (Suppl Table 3).

**Table 6:** Treatment Success and Failure Rates Across Increasing Score Categories

Treatment Outcome	Score 0	Score 1	Score 2	P-value
Success, n (%) Failure, n (%)	100 (97.1)^a^ 3 (2.90)	88 (78.6)^b^ 24 (21.4)	5 (50.0)^b^ 5 (50.0)	< 0.001

Groups with different superscript letters (a, b) indicate statistically significant differences in pairwise comparisons following chi-square analysis with Bonferroni correction

**Fig. 4:**
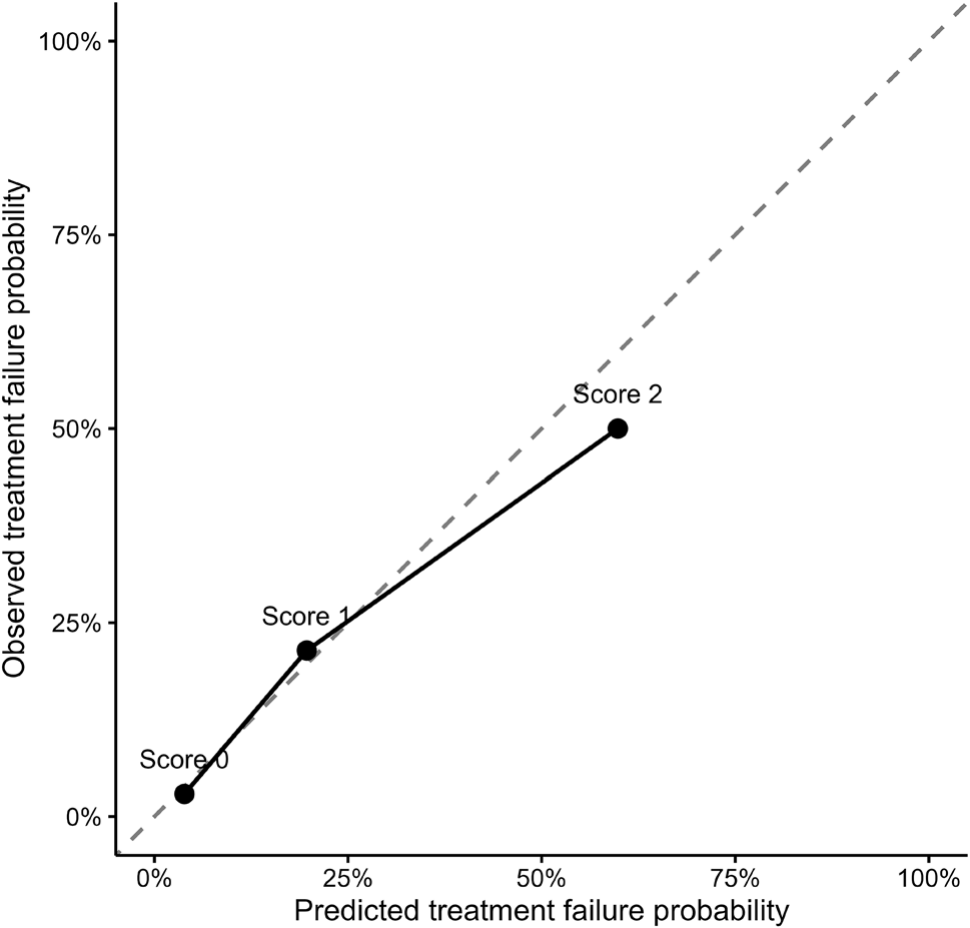
Calibration performance of the exploratory scoring model for prediction of treatment failure

To further evaluate the performance of the exploratory scoring model, calibration analyses were performed in addition to discrimination assessment. Overall predictive accuracy was supported by a Brier score of 0.108. Observed treatment failure rates were 2.9% (3/103), 21.4% (24/112), and 50.0% (5/10) for scores of 0, 1, and 2, respectively, while the corresponding predicted failure rates were 3.9%, 19.7%, and 59.8%. The calibration plot demonstrated a close correspondence between predicted probabilities and observed event rates across score categories. Furthermore, the Hosmer–Lemeshow goodness-of-fit test showed no evidence of significant miscalibration (χ^2^ = 0.875, df = 1, p = 0.350). Calibration intercept and slope were 0.00 and 1.00, respectively, indicating agreement between predicted and observed outcomes within the study cohort (Fig. 4).

## Discussion

### General findings and treatment success

This retrospective study evaluated MTX efficacy in patients with ectopic pregnancy and explored predictors of treatment failure in the overall cohort. MTX outcomes were compared between the indication and relative contraindication groups, with subgroup analyses within the latter. Overall, MTX success was 85.8%, and 65.5% in the relative contraindication group. High baseline β-hCG levels and pelvic free fluid ≥ 5 cm in the pouch of douglas were significantly associated with failure.

Reported MTX success rates in the literature range from 71% to 96.7% [1, 3, 5, 6, 11, 20–22], reflecting heterogeneity in populations, success definitions, and inclusion criteria. Some studies included patients with baseline β-hCG levels > 5000 mIU/mL or positive FCA, while others did not [1, 11, 20–22]. High reported success rates may be explained by defining failure only as cases requiring surgery, selecting patients with lower baseline β-hCG levels, smaller ectopic masses, or absent FCA, and differences in MTX timing. In our study, relative contraindication cases had lower success (65.5%) versus the indication group (92.8%). Nonetheless, a 65% success rate remains clinically meaningful. The absence of complications, close inpatient monitoring, and immediate surgical availability support MTX as a safe first-line option in these cases, potentially avoiding secondary complications from surgery and anesthesia use.

### Baseline β-hCG and MTX treatment outcome

In our study, baseline β-hCG levels were associated with MTX treatment failure in the multivariate analysis. This observation is in line with prior reports demonstrating an association between β-hCG levels and treatment outcomes [2, 5, 21, 23], although some studies have not identified a significant relationship [24, 25]. In addition, β-hCG levels > 910 mIU/mL during therapy have been linked to an increased risk of rupture [13]. In our cohort, the overall treatment success rate was 96.3%, with an optimal cutoff value of 1968 IU/L. Baseline β-hCG levels in patients with treatment failure were approximately 2.6-fold higher than in those with successful outcomes. Furthermore, each 1000 IU/L increase in β-hCG was associated with a 32% increase in the risk of treatment failure, with rates approaching 50% in cases with levels ≥ 4000 IU/L [5, 16]. Some studies have suggested reconsideration of the current guideline upper limit of 5000 IU/mL [5]. Reported cutoff values vary widely across studies (1500–9000, 2000–5000, and 6000–15,000 mIU/mL) [2, 26, 27], which may reflect differences in patient selection, treatment protocols, and surgical decision thresholds. In our study, the treatment success rate was 50% among patients with β-hCG levels ≥ 10,000 IU/L, further supporting the potential role of baseline β-hCG in predicting treatment outcomes. Dichotomization of baseline β-hCG at 1968 IU/L, as applied in the present analysis, may facilitate clinical interpretability and integration into the proposed risk stratification score; however, this approach may also be associated with some loss of information.

### β-hCG dynamics and MTX response

The prognostic value of β-hCG changes during MTX therapy remains uncertain. Early β-hCG variation (within 48 h before treatment) has shown variable and inconsistent predictive performance [13, 23, 26]. Some studies have highlighted the potential diagnostic value of changes between days 0/1 and 4 [9, 14, 26], whereas others have suggested that day-4 measurements may not be necessary [1, 20, 24, 28]. Transient hCG rises due to trophoblastic lysis, reported in approximately 26–60% of cases, may limit the accuracy of day 0/1–4 measurements [14, 26, 29]. Early intervention on day 4 may require careful consideration of potential risks in comparison to waiting until day 7 [1]. Some evidence suggests that day 0/1–7 monitoring may provide comparable predictive performance to day 4–7 protocols [20, 24, 30–32]. In single-dose MTX protocols, as described by Stovall, a ≥ 15% decline in β-hCG between days 4–7 is generally considered a standard criterion [9, 33]. In our study, β-hCG dynamics alone did not appear to be strong predictors of MTX treatment response; pretreatment increase rates and changes between days 0–4, 0–7, and 4–7 were not statistically significant. Day-4 β-hCG levels were observed to be higher in unsuccessful cases within the relative contraindication group.

### Pelvic free fluid

In our study, the presence of free fluid in the pouch of Douglas was significantly associated with MTX failure. This is consistent with prior reports indicating that pelvic fluid detected by TVS may be associated with tubal rupture, with some studies suggesting an increased risk of up to sixfold [3]. However, some studies did not confirm this association [2, 21], possibly because free fluid can result from tubal abortion as well as from rupture. Indeed, most surgically treated cases in previous reports (65%) demonstrated tubal abortion, and higher pelvic fluid has been reported to be associated with a reduced need for additional MTX doses [3, 34]. The widely reported incidence of rupture (20–77%) may partly reflect the misclassification of tubal abortion as rupture [3, 23, 35]. Accurate distinction between rupture and tubal abortion, careful ultrasonography, and serial β-hCG monitoring are therefore important considerations in relatively contraindicated cases. In the overall cohort of our study fluid accumulation ≥ 5 cm in the pouch of Douglas was significantly more frequent among MTX failures. Furthermore, in a descriptive analysis of patients with relatively contraindicated ectopic pregnancy, all surgically treated patients with pelvic free fluid accumulation ≥ 5 cm were found to have confirmed tubal rupture.

### Fetal cardiac activity and yolk sac

In our study, FCA was significantly associated with MTX failure in univariate analysis but was not retained as an independent predictor in the multivariate model performed in the overall cohort. The literature remains inconsistent, with some studies advocating surgery as a first-line approach, while others consider FCA a relative contraindication to MTX treatment [16, 26]. FCA has been reported to increase the risk of MTX failure by up to 12-fold [16]. Single-dose MTX success in FCA-positive cases has reached 88% in some studies, although double-dose regimens may be more effective [36, 37]. In our relative contraindication group, 50% of FCA cases responded to MTX, suggesting that FCA may represent a potential but not definitive risk factor. This discrepancy with prior studies may reflect the single-dose protocol, sample size limitations, and patient selection criteria. Similarly, the role of the yolk sac remains controversial: some studies, consistent with our findings, found no effect on MTX success [34], while others have reported it as an independent predictor of treatment failure [11, 16, 25, 26].

### Adnexal mass size

In the literature, adnexal masses > 3.5–4 cm are generally considered a relative contraindication for MTX, and have been proposed to be associated with more advanced disease [17, 37]. However, several studies have reported no significant association between mass size and treatment success, which has been attributed in some reports to measurement challenges related to surrounding hemorrhage [2, 14, 17, 21]. In our cohort, overall mass size was not significantly associated with MTX efficacy. Notably, in the relative contraindication group, MTX succeeded in 71.4% of cases with masses > 4 cm, which may suggest that mass size alone may not be a reliable predictor of outcome.

### Clinical factors and symptoms

The impact of maternal age and gravidity on MTX success remains controversial. Some studies reported no significant effect [2, 5, 21, 38], while others have reported that increasing age may be associated with lower success rates, with some studies suggesting approximately a twofold reduction per 10 year increase [6, 13, 16]. In our study, successful cases in the relatively contraindicated group were observed to be older. Vaginal bleeding, pelvic pain, and endometrial thickness were more frequent in the relatively contraindicated group, which is consistent with some reports suggesting a possible association between these factors and reduced MTX efficacy and increased rupture risk [7, 16, 35], although others found no association [17].

### MTX dose strategies

In the literature, multi- or two-dose MTX regimens have been reported to improve success rates in high-risk cases [35, 39] and may be considered for patients with β-hCG > 5000 IU/L, large adnexal masses, or an FCA [6, 21, 40, 41]. In our study, a single-dose MTX protocol was used based on clinical findings and patient preferences. Only 14% of patients received a second single-dose of MTX, 42% of whom were relatively contraindicated. Second-dose methotrexate administration was not randomly assigned but selectively given to patients with an inadequate initial response. This represents a clinically driven, response-based treatment strategy that may have influenced cohort composition, leading to non-random allocation and potential selection complexity. Consequently, this subgroup may represent a higher-risk population, contributing to potential selection bias and increased heterogeneity within the overall cohort. In our study, no statistically significant association was observed between the number of MTX doses and treatment success. To assess the potential impact of second-dose MTX administration, a sensitivity analysis restricted to single-dose MTX cases was conducted. The main factors associated with treatment failure, namely baseline β-hCG levels and pelvic free fluid ≥ 5 cm, showed similar findings across analyses regardless of MTX dosing strategy. Although single-dose MTX offers lower cost, fewer side effects, and easier monitoring [1, 6], the absence of a multi-dose comparison group limits the interpretation of these findings. Nevertheless, single-dose MTX is widely used as a first-line treatment in many centers, even for high-risk patients.

### Tubal rupture

Tubal rupture is one of the most common and serious complications of ectopic pregnancy, potentially causing hemodynamic instability and life-threatening hemorrhage. In our study, the overall tubal rupture rate after MTX therapy was 14.2%, in line with some reports [3, 13, 35], increasing to 34.5% in relatively contraindicated cases. Rupture may still occur in patients without identifiable risk factors [3, 23, 35]; in our cohort, this was observed in 7.2% of patients. Surgical intervention due to treatment failure typically occurred at a median of 7 days (range: 1–30 days) after MTX, similar to previous reports [3, 13, 35]. A decline in β-hCG levels was observed in approximately 70% of cases with rupture, suggesting that a decrease in β-hCG levels alone may not reliably exclude rupture [23, 42]. Therefore, β-hCG decline should not be considered as a sole indicator of safety, and close monitoring is required in all patients. These findings may suggest that the mechanisms underlying tubal rupture remain incompletely understood, highlighting the need for further research to better clarify risk factors and improve management.

### Scoring systems

Scoring systems and nomograms have been developed in the literature to predict MTX treatment outcomes using parameters such as initial β-hCG, mass size, yolk sac, FCA, and MTX dose number to identify high-risk cases [6, 10, 11, 43]. While these tools may be useful for risk stratification and individualized follow-up, their clinical applicability may be limited by variability in parameter assessment and study populations. High scores do not guarantee treatment failure but can help identify patients who require closer monitoring. In the multivariable logistic regression analysis performed in the entire study cohort, baseline β-hCG > 1968 IU/L and pelvic free fluid ≥ 5 cm were identified as independent predictors of MTX treatment failure. Based on these findings, a simple 0–2 point scoring system was constructed, assigning 1 point for each risk factor, to facilitate patient stratification according to treatment risk. Increasing score categories were associated with progressively lower treatment success, suggesting potential discriminatory performance of the model. However, this scoring system was derived from a single-center retrospective dataset and has not undergone external or prospective validation. Therefore, it should be considered an exploratory risk stratification tool rather than a clinically validated triage system and requires external validation before clinical implementation.

### Strengths and limitations

This single-center, retrospective study has limited generalizability. Variability in imaging measurements, heterogeneity in criteria such as β-hCG, mass size, and FCA, and differences in defining treatment success limit comparisons with the literature. Only a single-dose MTX protocol was used, and the lack of a multi-dose comparator group limits evaluation of potentially more effective regimens in high-risk patients. The multivariable logistic regression model was based on the entire study cohort, with an observed EPV of approximately 10.7, which is close to the lower boundary of commonly accepted levels and may affect the stability and generalizability of the model estimates. The number of events was relatively limited within the relatively contraindicated subgroup, further limiting subgroup-level inference. The proposed predictive scoring model was exploratory and should be interpreted with caution due to the retrospective design and limited number of events, and external validation is required before clinical application. A potential strength of the study is that sensitivity analysis in single-dose MTX patients showed similar findings to the primary model, with baseline β-hCG and pelvic free fluid ≥ 5 cm remaining associated with treatment failure. The number of MTX doses was not included as an independent predictor in the model because second-dose administration was applied selectively based on an inadequate initial response rather than as a baseline treatment strategy. This approach may reduce the risk of post-treatment bias. Additionally, the detailed analysis of patients with relatively contraindicated features and the evaluation of factors associated with treatment response may be considered strengths of the study. Compared with several previous studies, limited data are available on the separate analysis of this subgroup, and our findings may contribute to the existing literature.

## Conclusion

In carefully selected patients, methotrexate may represent an alternative to surgical management in ectopic pregnancies with relative contraindications. Baseline β-hCG levels and pelvic free fluid ≥ 5 cm in the pouch of Douglas were identified as independent risk factors for treatment failure. Management may benefit from a case-by-case approach considering β-hCG levels, Douglas fluid, adnexal mass size, and FCA. The proposed risk stratification model may help identify patients at higher risk of MTX failure; however, it requires external validation before clinical use. These findings may contribute to a more refined understanding of methotrexate selection criteria and support individualized treatment strategies. Future randomized trials including larger numbers of relatively contraindicated cases are needed to better assess the efficacy and safety of medical management in this population. Future research may also focus on identifying predictive biomarkers to optimize treatment selection and improve outcomes.

## Supplementary Information

Below is the link to the electronic supplementary material.

Supplementary file1 (DOCX 23 KB)

## Data Availability

No datasets were generated or analysed during the current study.
