# Supplementary material for: Methotrexate in relatively contraindicated ectopic pregnancies: who benefits from treatment?
Source: Arch Gynecol Obstet. 2026 Jul 11;313(1):239. doi: 10.1007/s00404-026-08501-4 (PMC13437762; doi:10.1007/s00404-026-08501-4)
Supplement: Supplementary file 1 [file 404_2026_8501_MOESM1_ESM.docx]

**Supplemental Table 1.** **Comparison of demographic, clinical and laboratory characteristics according to treatment outcome in single-dose MTX therapy**

| **Variable** | **Failure (n=26)** | **Success (n=168)** | **P-value** |
| --- | --- | --- | --- |
| Age (years) | 32.0 (25.0-34.0) | 32.0 (28.0-38.0) | 0.250 |
| Gravidity (n) | 3.00 (2.00-3.00) | 3.00 (2.00-4.00) | 0.408 |
| Gestational age (weeks) | 6.52 ± 1.44 | 6.09 ± 1.91 | 0.402 |
| CS history, n (%) | 10 (38.5) | 68 (40.5) | 0.845 |
| AB history, n (%) | 8 (30.8) | 62 (36.9) | 0.699 |
| EP history, n (%) | 5 (19.2) | 22 (13.1) | 0.372 |
| Mass size (mm) | 16.0 (0.00-21.0) | 12.0 (0.00-20.0) | 0.138 |
| Mass size ≥ 40 mm, n (%) | 2 (7.70) | 3 (1.80) | 0.134 |
| Fluid in Douglas (cm) | 1.00 (0.00-3.00) | 0.00 (0.00-1.00) | 0.011 |
| Fluid in Douglas ≥ 5 cm, n (%) | 5 (19.2) | 6 (3.60) | 0.008 |
| Endometrial thickness (mm) | 6.50 (6.00-10.0) | 6.00 (6.00-10.0) | 0.431 |
| Visible yolk sac, n (%) | 2 (7.70) | 6 (3.60) | 0.292 |
| Revision curettage, n (%) | 12 (46.2) | 71 (42.3) | 0.873 |
| Symptoms, n (%)  Vaginal bleeding  Pelvic pain  Vaginal spotting and pelvic pain  Asymptomatic | 9 (34.6)  8 (30.8)  6 (23.1)  1 (3.80) | 72 (42.9)  34 (20.2)  31 (18.5)  2 (1.20) | 0.562  0.338  0.594  0.352 |
| Positive fetal heartbeat, n (%) | 3 (11.5) | 3 (1.80) | 0.033 |
| Hemoglobin (g/dL) | 11.4 ± 1.37 | 11.6 ± 1.22 | 0.273 |
| Hematocrit (%) | 34.6 ± 3.77 | 35.2 ± 3.19 | 0.383 |
| LOS (days) | 8.00 (7.00-12.0) | 7.00 (4.00-10.0) | 0.035 |
| β-hCG Resolution Time (days) | 23.5 (18.0-29.0) | 24.0 (20.0-34.0) | 0.273 |
| Day-0 β-hCG (IU/L) | 5407 (2294-9881) | 1622 (515.0-3358) | <0.001 |
| Day-4 β-hCG (IU/L) | 4717 (2160-7825) | 957.0 (335.8-2389) | <0.001 |
| Day-7 β-hCG (IU/L) | 3194 (881.5-5114) | 647.5 (171.0-1800) | <0.001 |
| β-hCG change%: day-4 vs. day-0 | -1.99 (-36.8 to 26.2) | -16.9 (-44.5 to 5.66) | 0.149 |
| β-hCG change%: day-7 vs. day-4 | -39.9 (-54.3 to -25.8) | -31.4 (-56.6 to -17.6) | 0.447 |
| β-hCG change%: day-7 vs. day-0 | -45.5 (-70.3 to -2.12) | -45.2 (-74.8 to -16.6) | 0.550 |
| Rate of β-hCG rise in 48 hours  (pre-treatment) | 5.71 (-8.79 to 25.0) | 3.05 (-7.81 to 17.2) | 0.385 |

P<0.05: Statistically significant. CS: Cesarean Section, AB: Abortion, EP: Ectopic Pregnancy, MTX: Methotrexate, LOS: Length of Stay, β-hCG: Beta-Human Chorionic Gonadotropin.

| **Variables** | **Univariate (Unadjusted)** | | **Multivariate (Adjusted)** | |
| --- | --- | --- | --- | --- |
|  | **OR (95%CI)** | **P-value** | **OR (95%CI)** | **P-value** |
| Initial β-hCG > 1968 IU/L | 6.658 (2.199-20.16) | 0.001 | 5.999 (1.917-18.77) | 0.002 |
| Positive fetal heartbeat | 7.174 (1.366-37.68) | 0.020 | 3.488 (0.605-20.10) | 0.162 |
| Fluid in Douglas ≥5 cm | 6.429 (1.804-22.91) | 0.004 | 6.095 (1.497-24.82) | 0.012 |

**Supplemental Table 2. Binary logistic regression analysis for predictors of treatment failure in patients**

**receiving single-dose MTX therapy**

OR: Odds ratio, MTX: Methotrexate, β-hCG: Beta-Human Chorionic Gonadotropin.

**Supplemental Table 3. Receiver Operating Characteristic analysis the proposed scoring system for predicting MTX treatment failure.**

| **AUC** | **95 CI%** | **Cut-off** | **Sensitivity** | **Specificity** | **P-value** |
| --- | --- | --- | --- | --- | --- |
| 0.738 | 0.676 to 0.794 | ≥1 | 90.6% | 51.8% | **<0.001** |
